# A single-center initial experience on laparoscopic pancreatic operation combined with hepatic arterial resection and reconstruction

**DOI:** 10.3389/fsurg.2023.1153531

**Published:** 2023-05-17

**Authors:** Jie Xu, Jia-Guo Wang, Kai Lei, Zuo-Jin Liu

**Affiliations:** Department of Hepatobiliary Surgery, The Second Affiliated Hospital of Chongqing Medical University, Chongqing, China

**Keywords:** laparoscopic pancreas operation, laparoscopic technique, hepatic arterial resection and reconstruction, pancreatic ductal adenocarcinoma, arterial involvement

## Abstract

**Objective:**

This study aims to summarize our single-center initial experience in laparoscopic pancreatic operation (LPO) combined with hepatic arterial resection and reconstruction, as well as to demonstrate the feasibility, safety, and key surgical procedure for LPO.

**Methods:**

We retrospectively analyzed 7 patients who had undergone LPO combined with hepatic arterial resection and reconstruction in our center from January 2021 to December 2022. The clinical data of these 7 patients were collected and analyzed.

**Results:**

In our case series, two patients underwent passive arterial resection and reconstruction due to iatrogenic arterial injury, and five patients underwent forward arterial resection and reconstruction due to arterial invasion. The arterial anastomosis was successful in 5 cases, including 2 cases of end-to-end *in situ* and 3 cases of arterial transposition, and the vascular reconstruction time was 38.28 ± 15.32 min. There were two conversions to laparotomy. The postoperative recovery of all patients was uneventful, with one liver abscess (Segment 4) and no Clavien III–IV complications. We also share valuable technical feedback and experience gained from the initial practice.

**Conclusions:**

Based on the surgeon's proficiency in open arterial resection and reconstruction and laparoscopic technique. This study demonstrated the feasibility of total laparoscopic hepatic arterial resection and reconstruction in properly selected cases of arterial involvement or iatrogenic arterial injury. Our initial experience provides valuable information for laparoscopic pancreas surgery with arterial resection and reconstruction.

## Introduction

Pancreatic ductal adenocarcinoma (PDAC) has the 14th highest incidence and is the 7th leading cause of cancer-related death worldwide (1). The majority (approximately 80%) of patients are initially diagnosed with metastatic or locally advanced or borderline resectable status, and only approximately 20% of patients have prior opportunities for radical surgical resection (2). The 5-year overall survival rates of patients with resectable, locally advanced, and metastatic PDAC were 32%, 12%, and 3%, respectively, suggesting that tumor resection is directly related to patient prognosis (3). With neoadjuvant FOLFIRINOX (4–6) combined arterial or major venous resection (7, 8) applied to borderline resectable pancreatic cancer (BRPC) and locally advanced pancreatic cancer (LAPC), the overall radical resection rate of pancreatic cancer has been improved to some extent. With the rapid spread of standardized surgical procedures for pancreatic cancer and the optimization of perioperative patient management, increasing evidence supports pancreatic surgery combined with arteriovenous resection and reconstruction for LAPC patients with encouraging short-term and long-term prognoses (9–11).

Multiple pancreatic surgery centers have reported that minimally invasive pancreatic surgery can be performed safely and effectively by experienced pancreatic surgeons (12, 13). Due to its advantages of less trauma, faster recovery, less bleeding, and higher postoperative quality of life, it has gradually become the future development trend of pancreatic surgery (12, 14). In clinical practice, important factors that affect the number of minimally invasive pancreatic surgeries and the incidence of open conversion mainly include artery involvement or iatrogenic arterial vessel injury. There are few reports about laparoscopic pancreatic surgery combined with arterial resection and reconstruction. Therefore, the primary purpose of this study was to describe our initial experience in performing laparoscopic pancreatic surgery with arterial reconstruction.

## Methods

This study was an exploratory study approved by the ethics committee of the Second Affiliated Hospital of Chongqing Medical University, and written informed consent was obtained from each patient. From 2017 to 2022, our center performed 458 laparoscopic pancreatic surgeries for benign and malignant tumors. Of these, 368 patients underwent minimally invasive pancreatic surgery for malignant tumors [including laparoscopic pancreaticoduodenectomy, radical antegrade modular pancreatosplenectomy (RAMPS), and laparoscopic total pancreatectomy]. Our institution instituted a formal multidisciplinary tumor board for the treatment of new malignancies, and new malignancy cases were presented for decision-making and discussion.

All arterial resection and reconstruction surgeries were performed by the same surgeon. Based on the long-term practice of liver transplantation and minimally invasive pancreatic surgery, the surgeon is proficient in both open arterial resection and reconstruction techniques and laparoscopic techniques. Patients with solid tumor contact with the artery allowing for safe and complete resection and reconstruction or with iatrogenic damage to the artery that cannot be sacrificed were included in the study. We evaluated the resectability of pancreatic tumors and the length of arterial invasion by using multidetector computed tomography (MDCT), magnetic resonance imaging (MRI), and endoscopic ultrasonography before the operation. CT angiography (CTA) can be performed in specific cases, especially in the case of arterial invasion, which can more accurately evaluate the diameter of the two ends of arterial anastomosis and develop alternative arterial anastomosis schemes. We mainly selected laparoscopic hepatic arterial resection and reconstruction at the initial stage of laparoscopic resection and reconstruction. There are two main reasons for this. First, the surgeon has skilled experience in open hepatic arterial resection and reconstruction. Second, if there are complications after arterial anastomosis, we can remedy them through interventional surgery. Demographic characteristics, surgical procedures (arterial anastomosis schemes, estimated intraoperative blood loss, duration of operation, and duration of arterial reconstruction), and postoperative parameters (postoperative serum transaminase, postoperative hospital stay, and complications) were prospectively collected.

### The procedure of LPD combined with arterial resection and reconstruction

**Figure 1:**
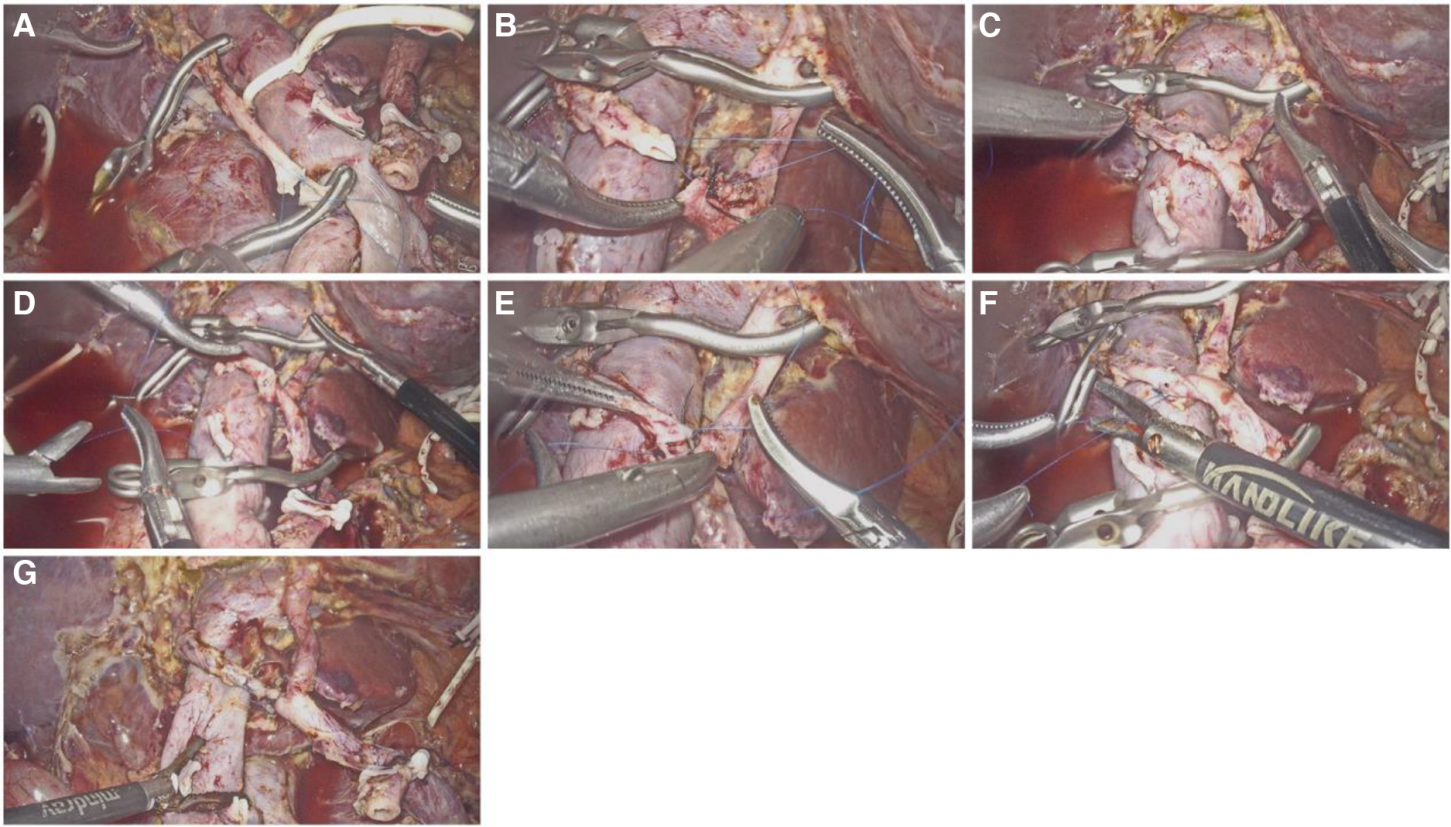
Arterial anastomosis process of rRHA and GDA. (**A**) In situ end-to-end anastomosis of the rRHA; (**B**) The distal end of rRHA was anastomosed with GDA, and suture began at 3 o'clock; (**C**) Continuous suture of the anterior wall from 3 o'clock to 9 o'clock; (**D**) Rotate the anastomosis 180°to turn the posterior wall into the anterior wall; (**E**) Continue to suture the posterior wall continuously; (**F**) The stitches of the anterior wall and the posterior wall converge and complete the knot; (**G**) No stenosis of the vessel after anastomosis and fluctuated well.

The procedure is performed using a five-trocar technique, and a 3D laparoscope (Karl Storz IMAGE1 S D3-Link™ and 10-mm TIPCAM®1S 3D passive polarizing laparoscopic systems) is used. The detailed process of arterial resection and reconstruction (arterial transposition) was as follows (Figure 1). (1) In situ end-to-end anastomosis of the replaced right hepatic artery (rRHA) was preferred, but it was found that the tension on both sides of the anastomotic end was too high to complete the anastomosis. (2) During the operation, the anastomosis plan was adjusted, and the stump of the gastroduodenal artery (GDA) with moderate tension and similar diameter was selected for reconstruction anastomosis. (3) The GDA stump was trimmed and the distal common hepatic artery (CHA) and proximal left hepatic artery (LHA) was temporarily clamped with the bulldog clamp. (4) For arterial anastomosis, a 5-0 H-S vascular suture was used to suture continuously from 3 o'clock to 9 o'clock. The vascular anastomosis was rotated 180° back to turn the posterior wall into the anterior wall. Suturing was continued from 3 o'clock to 9 o'clock until the anterior and posterior wall stitches converged and knotted to complete the anastomosis. (5) The temporary blocking forceps were released, and after careful examination, slight bleeding was found in the posterior wall at 4 o'clock, and a 5-0 prolene suture was applied to complete the repair. (6) The anastomosis was satisfactory after careful examination again, and the vessels were unobstructed and fluctuated well without bleeding or stenosis. After successful arterial anastomosis, bilioenterostomy and gastrointestinal anastomosis were performed. (See Supplementary Video S1, Supplemental Digital Content 1).

**Figure 2:**
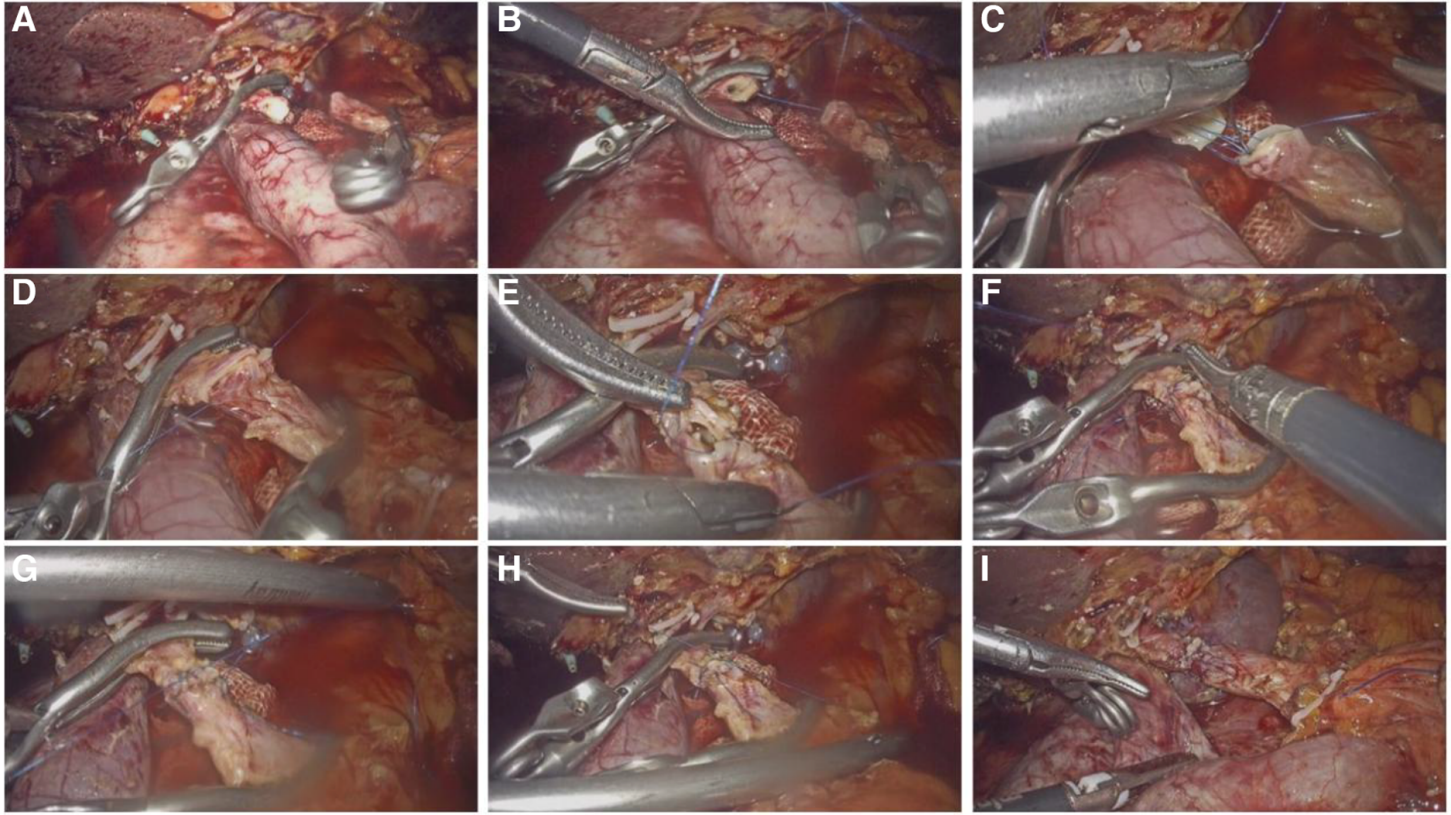
Arterial anastomosis process of CHA end-to-end *in situ*. (**A**) The both ends of the anastomosis were reshaped; (**B**) Suture began at 3 o'clock; (**C**) Continuous suture of the posterior wall of the blood vessel; (**D**) Completion of continuous suture on the posterior wall; (**E**) Continue to suture the anterior wall; (**F**) Completion of continuous suture on the posterior wall; (**G**) The stitches of the anterior wall and the posterior wall converge and complete the knot; (**H**) Strengthen the weak position of the anastomosis; (**I**) No stenosis of the vessel after anastomosis and fluctuated well.

The detailed process of end-to-end arterial resection and reconstruction (Figure 2) is similar to the surgical procedure of arterial transposition described above (See Supplementary Video S2, Supplemental Digital Content 2).

The detailed process of end-to-end arterial resection and reconstruction (Figure 2) is familiar with the surgical procedure of arterial transposition described above. (See Supplementary Video 2, Supplemental Digital Content 2).

### Postoperative management

With the introduction of enhanced recovery after surgery (ERAS), all patients who underwent arterial anastomosis were given perioperative prophylactic antibiotic therapy and their plasma albumin levels were maintained above 35 g/L. Due to the possibility of ischemia‒reperfusion injury after arterial occlusion, low-dose glucocorticoid therapy was administered 3 days after surgery. All patients were given LMW heparin at 2,000 iu/day except for those with abdominal bleeding. The gastric tube was usually removed, and the patient was encouraged to drink a small amount of water on the first postoperative day. The patient was instructed by a clinical dietitian to begin enteral nutrition on the third postoperative day. The level of ascites amylase was detected every day for the first 5 days after surgery, and enhanced abdominal CT was reviewed 3–5 days after surgery. The abdominal drainage tube could be pulled out if there was no obvious abdominal effusion and if the ascites amylase level was lower than 1,000 IU/l. Abdominal CT should further determine the patency of the artery anastomosis and rule out complications related to liver ischemia and false aneurysm. Postoperative pancreatic fistula (POPF) (15), delayed gastric emptying (16), and postoperative complication grading (17) are relevant international standards.

All patients began adjuvant therapy within 8 weeks to 12 weeks after surgery. Abdominal imaging examinations were reviewed every 3 months after surgery.

### Statistical analysis

Descriptive statistics were used for evaluating variants. Arterial anastomosis time, operation time, postoperative transaminase level, vesicle length, etc., are expressed as the mean ± standard deviation, and the age, BMI, postoperative hospital stay, and number of lymph nodes harvested are presented as the median and interquartile range. Categorical data are expressed as numbers and percentages. SPSS version 22.0 (SPSS Inc., IBM, Armonk, NY, USA) was used for all analyses.

## Results

A total of 7 patients were included in this study, including 5 males and 2 females. Table 1 summarizes the patient characteristics and surgical details. The median age of the patients was 62 years. The median BMI was 24.6 kg/m^2^. Pathological diagnoses included 5 cases of PDAC, 1 case of lower biliary duct carcinoma, and 1 case of NET. There was only 1 case of ASA status III/IV. Five patients underwent preoperative neoadjuvant therapy. 7 patients underwent laparoscopic pancreatic surgeries with arterial resection and reconstruction (three laparoscopic total pancreatectomy, four laparoscopic pancreaticoduodenectomy). Of these, two patients underwent passive arterial resection and reconstruction due to iatrogenic arterial injury, and five patients underwent forward arterial resection and reconstruction due to arterial invasion. The vesicle length was 2.6 ± 1.54 cm due to tumor invasion. The operative time was 380.67 ± 125.34 min, the mean vascular reconstruction time was 38.28 ± 15.32 min, and the mean intraoperative blood loss was 520 ml (410–800 ml). There were two conversions to laparotomy, and the median postoperative hospital stay was 18 days (14–25 days). There were no Clavien III-IV complications. Postoperative pancreatic fistula consisted of two A fistulas and one B fistula. Among the ischemic complications after arterial anastomosis, only 1 patient developed liver abscess (Segment 4) during postoperative hospitalization, which recovered after puncture and drainage. One patient was readmitted to the hospital for surgical repair due to perforation of a gastrointestinal anastomotic ulcer. The other patients obtained good short-term prognoses after surgery. There were totally 3 and 4 cases undergoing end-to-end and arterial transposition, respectively. The arterial anastomosis was successful in 5 cases, including 2 cases of end-to-end in situ and 3 cases of arterial transposition (Table 2). Main failure reasons include: 1) high tension of anastomotic end; 2) The artery diameter is too small (< 5mm); 3) Arterial anastomotic diameter mismatch (> 1.5 times).

**Table 1:** The demographic data of all 7 patients.

Demographic data	Frequencies	Range/Percentage
Sex		
Male	5	71.4%
female	2	28.6%
Age (year)	62	51–69
BMI (kg/m^2^)	24.6	22.3–26.7
American Society of Anesthesiology		
I	5	71.4%
II	1	14.3%
III	1	14.3%
Neoadjuvant therapy	5	71.4%
Tumor type		
PDAC	5	71.4%
NET	1	14.3%
Lower biliary duct carcinoma	1	14.3%
Type of pancreatectomy		
LTP	3	42.9%
LPD	4	57.1%
Arterial anastomosis time (min)	38.28 ± 15.32	31–59
Operation time (min)	380.67 ± 125.34	310–580
Vasculectomy length (cm)		
invasion	2.6 ± 1.54	2.1–3.4
iatrogenic injury	0	0
Intraoperative bleeding (ml) (ml)	520	410–800
Conversion to laparotomy	2	28.6%
Postoperation ALT/AST		
ALT(IU/L)	346 ± 150.68	208–545
AST(IU/L)	470 ± 230.22	223–724
Clavien III–IV complication	0	0
Postoperative pancreatic fistula		
A	2	28.6%
B	1	14.3%
C	0	0
Postoperative hospital stays (d)	18	14–25
Lymph nodes harvested	18	16–27

PDAC, pancreatic ductal adenocarcinoma; NET, neuroendocrine tumor; LTP, laparoscopic total pancreatectomy; LPD, laparoscopic pancreatoduodenectomy.

**Table 2:** Arterial anastomosis classified by fashions.

Fashion of arterial anastomosis	Outcome	Artery	Main failure reasons
End-to-end *in situ* (*n *= 3)	Success (*n *= 2)	rRHA (*n* = 1)	
		CHA(*n* = 1)	
	Failure (*n *= 1)	rRHA (*n* = 1)	The tension on both sides of the anastomotic end is too high and the arterial diameter is small (<5 mm).
Arterial transposition(*n* = 4)	Success (*n *= 3)	rRHA and GDA (*n* = 2)	
		CHA and SA(*n* = 1)	
	Failure (*n *= 1)	CHA and SA (*n* = 1)	the diameters of the two sides of the anastomosis end do not match (>1.5 times).

rRHA, replaced right hepatic artery; CHA, common hepatic artery; GDA, gastroduodenal artery; SA, splenic artery.

## Discussion

To the best of our knowledge, there are no relevant reports on pancreatic surgery combined with hepatic artery resection and reconstruction. In our cohort, an initial attempt was made at laparoscopic arterial resection and reconstruction and the initial experience was described while achieving encouraging short-term outcomes.

Overall, pancreatic cancer with arterial involvement is strongly associated with a worse oncologic prognosis. However, surgical treatment is still the only potential treatment option for patients with pancreatic ductal adenocarcinoma that can help prolong their survival time or long-term survival (18). Some early meta-analyses found that the overall short-term outcomes and oncological outcomes of pancreatectomy patients with artery resection were disappointing (19). With the advent of modern multimodal treatment with combination chemotherapy and radiotherapy, the overall prognosis for patients with PDAC has been greatly improved (20, 21). With the introduction of FOLFIRINOX's neoadjuvant treatment for pancreatic cancer, surgical resection rates for pancreatic cancer have improved somewhat and achieved encouraging survival data (22, 23). Safety and long-term oncological outcomes in LPD patients with arterial resection and reconstruction are of critical concern. Bachellier et al. reported that patients who underwent arterial resection had a median survival of 13.7 months (11–18.5 months) and a 5-year survival rate of 11.8% in their single center (24). Department of General, Visceral and Transplantation Surgery, Heidelberg University Hospital, also recently confirmed the safety and feasibility of arterial resection and reconstruction for locally advanced pancreatic cancer and reported a favorable overall survival rate (10). With the current minimally invasive process of pancreatic surgery, it is of great practical significance to explore a safe and effective method of laparoscopic pancreatic surgery with arterial resection and reconstruction.

There were the following main difficulties in the initial laparoscopic arterial resection and reconstruction: (1) how to choose between arterial resection with no reconstruction and arterial resection with reconstruction; (2) how to effectively deal with the problem of anastomotic diameter mismatch at both ends of arterial anastomosis; (3) how to effectively shorten the time of arterial anastomosis to avoid the effect of too long arterial anastomosis time on organ function; and (4) how to ensure that all three layers of the artery wall are sutured (especially the intima must be sutured) to avoid catastrophic complications (such as false aneurysms, post pancreatectomy hemorrhage, etc).

In view of the above technical difficulties, we summarize our initial experience in laparoscopic pancreatic surgery with arterial resection and reconstruction as follows. Complete preoperative imaging data were necessary (including artery involvement length, diameters at both ends of the anastomosis, whether there is collateral circulation on both sides of the anastomosis, etc.), and surgical plans and alternatives (including arterial anastomosis method, surgical approach, etc.) were formulated. In the early stage of laparoscopic arterial resection and reconstruction, the main object was the hepatic artery with a diameter greater than 5 mm. For arterial vascular anastomosis with an artery diameter of less than 5 mm and no alternative hepatic artery blood supply, laparotomy was preferred. When the diameters of the two sides of the anastomosis end did not match, if the difference was less than 1.5 times, the anastomosis could have been completed by properly adjusting the needle distance. If it was more than 1.5 times, arterial anastomosis was recommended after prioritizing arterial plastic surgery or direct laparotomy for anastomosis. Intraoperatively, the advantages of 3D laparoscopic systems for minimally invasive pancreatic surgery were reported, especially in LPD with simultaneous venous resection (25, 26). Therefore, to ensure that each stitch was performed under direct vision, all minimally invasive pancreatic surgery with vascular reconstruction was performed in 3D laparoscopy. The artery-first approach (supracolic right posterior, infracolic anterior, supracolic anterior, and infracolic left posterior) has been shown to improve overall R0 resection in pancreatic ductal adenocarcinoma (27).

Loos et al. also demonstrated that an arterial surgical approach is effective in achieving arterial resection in locally advanced pancreatic cancer with promising long-term survival (10). In our series, the artery-first approach was selected for all cases, and the selection of a specific approach was individualized according to tumor location, size, and the degree of artery involvement. We mainly used the supracolic right posterior approach and the anterior approach for hepatic artery anastomosis. The supracolic right posterior approach can help achieve good visualization of the SMA root, and right semicircular dissection of all soft connective tissues around the SMA can be implemented preferentially. We then took advantage of the anterior approach to dissociate the specimen and obtain favorable exposure of the arterial anastomosis. After complete resection of the specimens, we occluded blood flow and performed arterial resection and reconstruction. This approach can considerably shorten the duration of organ ischemia and reduce ischemia-related complications. Recently, several centers have reported robotic pancreaticoduodenectomy combined with venous resection and reconstruction (28, 29). These reports have all confirmed that robotic pancreaticoduodenectomy with venous resection and reconstruction is a feasible approach for selected patients with venous invasion, but they also strongly suggest that pancreaticoduodenectomy with venous resection and reconstruction should be performed by expert surgeons at high-volume centers. Although there are no reports of robot-assisted arterial resection reconstruction, with its unique advantages of intracorporeal sutures (30), it is conceivable that robotic pancreaticoduodenectomy combined with artery resection and reconstruction can be the next technological revolution in pancreatic surgery.

In general, laparoscopic arterial anastomosis is still the “bottleneck” of minimally invasive surgical techniques in the pancreas, and it is technically difficult to improve the minimally invasive resection rate in pancreatic cancer, especially in these patients after neoadjuvant chemotherapy. Prior to planned implementation of the procedure, the technical reserve of laparoscopic venous reconstruction technology and open arterial reconstruction technology is necessary. We recommend a team of fixed-skilled laparoscopic technicians with effective teamwork to complete the procedure.

However, the biggest limitation of this study is the small sample size. At present, laparoscopic arterial reconstruction is still in the early stage. We have only completed the two forms of arterial anastomosis, end-to-end anastomosis and arterial transposition, and have not tried interposition grafts yet. Another limitation is that currently, the hepatic artery is still the main reconstruction object, and reconstruction of the superior mesenteric artery (SMA) and celiac axis (CA) is still completed under laparotomy. We hope to gradually transition to the reconstruction of SMA and CA through more primary experience of hepatic artery resection and reconstruction in the future. It is worth noting that most cases have a good short-term prognosis, but long-term complications are unknown. Therefore, we need to further collect related cases and follow up on these patients to determine the long-term prognosis of laparoscopic arterial anastomosis.

## Conclusion

Laparoscopic pancreatic surgery combined with hepatic artery resection and reconstruction is safe and feasible. In addition, 3D laparoscopy can provide hardware support for arterial anastomosis. The “artery-first” approach, as the preferred approach for laparoscopic arterial anastomosis, has significant advantages in promoting the surgical process, reducing artery occlusion time, and improving the success rate of arterial anastomosis. It was also confirmed that the short-term prognosis of this technique is encouraging. However, the safety and feasibility of laparoscopic SMA anastomosis and CA anastomosis should be further explored in the future. The long-term prognosis of this technique also needs to be further confirmed by a larger sample size and long-term follow-up.

## Data Availability

The raw data supporting the conclusions of this article will be made available by the authors, without undue reservation.
